# The Diagnostic Utility and Clinical Implications of Bronchoalveolar Lavage in Cancer Patients With Febrile Neutropenia and Lung Infiltrates

**DOI:** 10.7759/cureus.10268

**Published:** 2020-09-06

**Authors:** Usman Khalid, Muhammad J Akram, Faheem M Butt, Mohammad B Ashraf, Faheem Khan

**Affiliations:** 1 Internal Medicine, Shaukat Khanum Memorial Cancer Hospital and Research Centre, Lahore, PAK

**Keywords:** bronchoscopy, chemotherapy-induced neutropenia, pneumonia, bal

## Abstract

Introduction

Febrile neutropenia (FN) is a dreaded complication of cancer chemotherapy and frequently associated with respiratory infections. Flexible bronchoscopy (FB) serves as a useful diagnostic tool in this regard.

Objective

To determine the diagnostic yield, safety and clinical implications of bronchoalveolar lavage (BAL) in cancer patients with FN, having lung infiltrates on radiographic chest imaging.

Methods

We reviewed medical records of FN patients who underwent FB at Shaukat Khanum Memorial Cancer Hospital and Research Centre, Lahore, from July 2015 till July 2018. The culture yield of BAL, resultant change of management and outcome over the subsequent 30 days were retrospectively analysed. Statistical Package for Social Sciences (SPSS) version 20 (IBM Corp., Armonk, NY) was used for data analysis.

Results

Ninety FN patients, with mean age 26 ± 18 years and predominantly males (65.6%, n = 59) were included in the study. Seventy-seven (85.6%) had hematological and 13 (14.4%) solid organ malignancy. The mean absolute neutrophil count was 0.20 +/- 0.36/ µL. BAL cultures were diagnostic in 40 (44%) patients; the etiology was bacterial, fungal and mixed in 25 (62.5%), 14 (35%) and one (2.5%) patient, respectively. All patients were on empirical antibiotics prior to bronchoscopy: 32 (35.6%) on antibacterial alone and 58 (64.4%) on antibacterial plus antifungal therapy. Change of management occurred in 51 (56.7%) patients after BAL results, including de-escalation from dual antibiotics in 28 (55%) and initiation of new culture sensitive antibiotic in 23 (45%). FB-associated complications developed in three (5.6%) non-intensive care patients (ICU), including transient hypoxia in two and minor hemoptysis in one patient, while five (14.8%) mechanically ventilated patients in ICU experienced worsening of oxygenation parameters within 48 hours. Overall, 24 (26.7%) patients died. Mortality was 3.7% in non-ICU and 69% in ICU setting and significantly higher in patients with fungal pneumonias (p-value 0.01) and with prolonged neutropenia (p-value 0.001).

Conclusions

BAL is a safe diagnostic tool for FN patients with lung infiltrates, with minimal complications and sufficient diagnostic yield to improve diagnosis and management of such patients.

## Introduction

Febrile neutropenia (FN), a dreaded complication of chemotherapy and cancer, is attributed with increased hospitalization costs, morbidity and mortality in cancer patients [1,2]. Respiratory infections are the leading cause of mortality among such patients, with a reported incidence of between 25-30% [2]. An empirical approach for antibiotic therapy is recommended for such patients, and immediate initiation of treatment supplants all diagnostic workup [3-5]. Nevertheless, an early etiological diagnosis is pivotal to improving overall management and outcomes. Bronchoalveolar lavage (BAL) using flexible bronchoscopy (FB) is regarded as a reliable diagnostic tool in patients with FN having lung infiltrates, with a reported diagnostic yield of around 22-65% and minimal complication risk [6-12].

Despite high diagnostic yield, opinion on the implications of bronchoscopy for FN patients remains divided. Certain authorities report high mortality with no significant improvement in outcome [13] while others convincingly advocate early intervention [14]. Wahla et al. revealed a significant disparity in viewpoints of treating physicians regarding the timing, necessity and plausibility of bronchoscopy in neutropenic patients [15]. Guidelines recommend early intervention, however, caution is advised due to concerns for high mortality in critically ill patients [16]. In order to justify benefit, it is pertinent to understand the clinical implications in terms of management change and not just diagnostic yield, as well as to correlate mortality with the overall clinical picture.

While working at a facility dedicated to cancer patients, we encounter both adult and pediatric patients who develop FN during the long haul of their treatment course. The purpose of this study was twofold; firstly, to determine the diagnostic culture yield and complication risk of BAL for FN patients in our population setting, and secondly to evaluate its implications on such patients in terms of change of empirical to targeted antibiotic therapy and the resultant outcomes in terms of mortality and discharge. One of the concerns that led to the need for such a study in our setup was the high mortality in FN patients despite timely institution of empirical treatment. With this endeavor, we intend to improve evidence-based practice in our setup and improve the quality of patient care.

## Materials and methods

After formal approval from the Institutional Review Board (IRB) of Shaukat Khanum Memorial Cancer Hospital and Research Center, Lahore, we acquired the data of 523 patients who underwent flexible bronchoscopy from July 2015 until July 2018. Of these, all patients with FN who underwent flexible bronchoscopy for diagnosis of lung infiltrates were shortlisted. The relevant clinical information was collected from the electronic medical records of the patients. Study design was observational (retrospective-cross sectional).

Neutropenia was defined as an absolute neutrophil count (ANC) of less than 500/µl, or less than 1000/µl with an expected decline to <500/µl in the subsequent 48 hours [17]. Febrile neutropenia was defined as fever in a neutropenic patient, recorded as a single oral recording of 38.3° C (101° F) or a temperature greater than 38.0° C (100.4° F) sustained for more than 1 hour [17]. Lung infiltrates were defined as development of new focal or diffuse pulmonary opacification, ground glass haziness and/or nodularity on CT chest, suggestive of infectious process.

Bronchoalveolar lavage (BAL) using flexible bronchoscopy had been obtained by injecting aliquots of 60 to 100 ml isotonic normal saline in the identified lung sub segment(s) followed by active suctioning. The specimen underwent Gram/Giemsa staining, bacterial and fungal cultures, Mycobacterium tuberculosis culture and rapid polymerase chain reaction (PCR). Additionally, silver staining for Pneumocystis jirovecii pneumonia (PJP) and AFB staining for Nocardia was performed wherever indicated. Positive cultures were diagnostic for bacterial and fungal pneumonias while in selected cases, elevated serum fungal markers {(1,3)-β-D Glucan and Aspergillus galactomannan} were regarded as diagnostic [18].

Patient medical records were followed for 30 days post procedure for any change of treatment. Change of treatment was defined as either switching over to a culture sensitive antibiotic or de-escalation of antibiotics to single genre where dual therapy was started empirically. Complications in the immediate 48 hours post procedure were noted. All data was analyzed in SPSS (Statistical Package for Social Sciences) version 20 (IBM Corp., Armonk, NY). Independent t- test was used to compare difference of means and p-value < 0.05 was considered significant.

## Results

A total of 90 patients with FN underwent bronchoscopy and BAL within the defined period, including 59 (65.6%) male patients, with mean age 26 ± 18 years. Out of total, 36 (40%) patients were admitted to intensive care unit (ICU), while 27/36 (75%) were maintained on mechanical ventilation prior to the procedure. Nineteen (21%) patients had a positive blood culture while sputum cultures were positive in just three (3.3%) patients. Among mechanically ventilated patients, tracheal aspirate cultures were positive in 6/27 (22.2%) patients. All patients had been started on empirical antibiotics, out of which 58 (64.4%) were on combination antibacterial and antifungal therapy (see Table 1 for demographic details).

**Table 1:** Key demographic variables of febrile neutropenic patients undergoing bronchoscopy shown as percentage or mean + standard deviation

Variables	Number (%)
Gender	
Male	59 (65.6%)
Female	31 (34.4%)
Mean age (years + SD)	26 ± 18
Mean absolute neutrophil count (per µL)	0.20 ± 0.36
Primary malignancy	
Leukemia	61 (67.8%)
Lymphoma	16 (17.8%)
Solid organ malignancy	13 (14.4%)
Radiological Impressions (CT Chest)	
Focal (Lobar) consolidation	26 (28.9%)
Diffuse consolidation / nodularity	64 (71.1%)
Patients with other positive cultures	
Sputum culture	03 (3.3%)
Blood culture	19 (21%)
Tracheal aspirate culture (in mechanically ventilated patients)	6/27 (22.2%)
Serum Fungal Markers : (1,3)-β-D Glucan and Aspergillus galactomannan	
Performed	78
Elevated	24/78 (31%)
Empirical antimicrobials	
Antibacterial alone	32 (35.6%)
Antibacterial + Antifungal	58 (64.4%)
Mean duration of admission before bronchoscopy (days + SD ^ǂ ^)	11 ± 7.90
Mean duration of empirical antibiotics (days + SD ^ǂ ^)	14 ± 7.95
Mean duration of neutropenic fever prior to procedure (days + SD ^ǂ ^)	10.26 ± 6.8
Duration of neutropenia (days)	
Up to 10 days	56 (62.2%)
More than 10 days	34 (37.8%)
Pre procedure Sedation / Anesthesia	
Short acting IV sedation	54 (60%)
General Anesthesia	36 (40%)

BAL cultures were positive and diagnostic in 40 (44%) patients and the etiology was bacterial, fungal and mixed in 25 (62.5%), 14 (35%) and one (2.5%) patients, respectively. The culture etiology was thus predominantly bacterial and Pseudomonas was identified as the most common etiology of lung infiltrates in our patients, followed by Aspergillus (as shown in Table 2).

**Table 2:** Etiology of Bronchoalveolar lavage (BAL) culture in patients with febrile neutropenia who underwent bronchoscopy

Etiology	Number
Bacterial	25/40 (62.5%)
Pseudomonas	10
Staphylococcus aureus	04
Stenotrophomonas	04
Mycobacterium tuberculosis	03
Enterococci	02
Burkholderia	01
Klebsiella	01
Fungal	14/40 (35%)
Aspergillus	08
Rhizopus	03
Pneumocystis jirovecii (PJP)	02
Candida	01
Mixed (bacterial + fungal)	01 (2.5%)
Staphylococcus aureus + Aspergillus	01 each

The final diagnosis was revised in patients with negative BAL culture as follows: The diagnosis of fungal pneumonia was retained in 12 (24%) such patients, based on radiological impressions and supported by raised 1, 3-β-D Glucan and Aspergillus galactomannan antigen levels. In the remaining culture negative patients, neutropenic sepsis was the final diagnosis in 11 (22%), neutropenic colitis and acute respiratory distress syndrome in six (12%) patients each, drug-induced pulmonary toxicity and pulmonary edema in five (10%) each, diffuse alveolar hemorrhage in three (6%) and pulmonary embolism and hypersensitivity pneumonitis in one (2%) patient each.

Subsequent to culture results, 51 (56.7%) patients underwent treatment modification. The change of treatment included de-escalation from dual combination (antibacterial + antifungal) to single genre in 28 (55%) and initiation of a new culture sensitive antibiotic in 23 (45%) patients overall. Change of treatment affected 34 (85%) with positive and 17 (34%) patients with negative BAL cultures. Eleven patients were determined to have sensitivity to an oral antimicrobial and hence parenteral treatment was discontinued. The duration of empirical antimicrobial treatment was determined to be longer in BAL culture negative patients for a median of two days compared to culture positive patients (interquartile 1, 32 days versus interquartile 1, 30 days), however the difference of means was statistically non-significant (p-value 0.4). Following the procedure, 66 (73.3%) patients were discharged within 30 days after necessary antibiotic modification, including 27 patients with positive and 39 patients with negative BAL cultures. BAL culture driven treatment modification affected 76.5% patients discharged.

Only three (5.6%) non-ICU patients reported complications within 48 hours of bronchoscopy. These included dyspnea/hypoxia in two and hemoptysis in one patient(s) respectively, both complications being self-resolving in the subsequent 24-48 hours. All 27 mechanically ventilated patients had baseline fraction of inspired oxygen (FiO2) requirement of ≤50%. Post procedure deterioration in oxygenation and ventilation parameters (which included increase in FiO2 and positive pressure requirement in all) was seen in at least four (14.8%) and a similar number died within five days of bronchoscopy. Only one non-ICU patient required mechanical ventilation after 72-96 hours of bronchoscopy, having worsening hypoxia and radiographic infiltrates, with dual etiology growth on BAL culture, and succumbed to acute respiratory distress syndrome on 2nd ICU and 6th post procedure day. Overall, 24 patients died, bringing the cumulative mortality to 26.7%. The mortality was 69% in ICU, 3.7% in non-ICU patients (see Table 3) and 55.6% for mechanically ventilated patients.

**Table 3:** Comparison between patients admitted to intensive care unit (ICU) and non-ICU setting in terms of duration of neutropenia, culture yield and outcome

Variable	Patients admitted to non-ICU setting	Patient admitted to ICU setting
Total	54	36
Duration of neutropenia (days ± SD^ǂ^)	8.22 ± 4.7	13.3 ± 8.3
Culture positive	24 (44.4%)	16 (44.4)
Bacterial	19	06
Fungal	04	10
Mixed	01	0
Mortality	2/54 (3.7%)	25/36 (69%)
Change of treatment	34/54 (63%)	18/36 (50%)

The mortality was significantly higher overall in patients with fungal pneumonia (57.1%, p-value 0.01) and those with longer duration of neutropenic fever (p-value 0.001). There also appeared to be a higher mortality with prolonged duration of hospitalization as well as empirical antibiotic therapy. There was no statistically significant mean difference of mortality between gender, cancers of different origin and age group (adult versus pediatric). See Table 4 for descriptive statistics of these outcome variables.

**Table 4:** Descriptive statistics of outcome variable with respect to explanatory variables in febrile neutropenic patients undergoing bronchoscopy

Variables Categories	Total	Discharged 66 (73.3%)	Mortality 24 (26.7%)	p-value
Gender				0.52
Male	59	42 (71.2)	17 (28.8)	
Female	31	24 (77.4%)	7 (22.6)	
Age				0.43
Adult (> 15 years)	62	47 (75.8)	15 (24.2)	
Pediatric (< 15 years)	28	19 (67.9)	9 (32.1)	
Type of Cancer				0.72
Leukemia	61	43 (70.5)	18 (29.5)	
Lymphoma	16	13 (81.2)	03 (18.8)	
Solid organ malignancy	13	10 (76.9)	03 (23.1)	
BAL CULTURE				
Negative	50	39 (78.0)	11 (22)	0.26
Positive	40	27 (67.5)	13 (32.5)	
Bacterial	25	21 (84)	4 (16)	0.01
Fungal	14	6 (42.9)	8 (57.1)	
Mixed	01	0	1 (100)	
Change of treatment				0.44
No	39	27 (69.2)	12 (30.8)	
Yes	51	39 (67.5)	12 (23.5)	
Mean duration of admission (days ± SD ^ǂ^)	11 ± 7.90	9.89 ± 7.22	13.66 ± 9.13	0.04
Mean duration of empirical antibiotics (days ± SD ^ǂ^)	14 ± 7.95	13.03 ± 7.19	17.08 ± 9.29	0.03
Mean duration of neutropenic fever (days ± SD ^ǂ^)	10.26 + 6.8	8.44 ± 5.16	15.86 ± 8.32	0.001

Significant co-morbidities complicated the clinical course in all of the 24 patients who died. Twenty such (83%) patients had severe neutropenic sepsis (11 with negative bronchial washing cultures). The main complications included septic shock in 18 (75%), multi organ dysfunction in eight (33%), disseminated intravascular coagulation in six (25%), acute respiratory distress syndrome (ARDS) in six (25%) and acute renal failure in 11 (46%) patients overall. Additionally, eight (33%) patients had severe anion gap metabolic acidosis while three (13%) developed hypothermia in terminal days. Five (21%) patients succumbed to upper gastrointestinal bleeding, three (13%) to pulmonary edema and heart failure secondary to drug-induced cardiotoxicity, two (8%) patients to intracranial hemorrhage, one (4%) patient to pulmonary embolism. Among the two non-ICU patients who died, the cause of death was severe upper gastrointestinal (GI) bleeding in one and hypokalemia-induced cardiac arrhythmia in second patient, both events not associated with bronchoscopy.

## Discussion

This retrospective study reports an appreciable diagnostic culture yield of BAL for patients with FN and lung infiltrates (44%), with minimal complication risks. The findings are comparable to previous studies in terms of diagnostic yield and safety [6-11]. The cumulative mortality in our study was 26.7%, however, the study elaborates that complications directly attributable to bronchoscopy were minimal. BAL culture result-driven therapeutic change affected 51 (56.7%) patients in total, including 63% non-ICU and 50% ICU patients, and the majority of patients (76.5%) who were subsequently discharged within 30 days. Thus, while certain previous studies may have reported that culture results do not benefit patients sufficiently in terms of any change in treatment [11,14], results of our study were otherwise. Against certain studies reporting reduced BAL culture yield with prolonged empirical antibiotic therapy [11,19,20], our study determines this association to be statistically non-significant.

As per our findings, the duration of neutropenic fever adversely affects mortality. Previously Seneviratna et al. [8] and Peikert et al. [10] had stated that such a relationship was non-significant. There was a statistically significant relation of prolonged duration of hospitalization and empirical therapy to mortality in our study. Drawing parallels, Rano et al. had reported a reduction in mortality in patients who underwent treatment change within seven days of hospitalization compared to those in whom it was delayed [21]. Mortality was also higher in BAL culture positive compared to culture negative patients in our study, which may correlate infection to mortality. Thus, the findings of our study imply that early intervention and culture results may improve outcomes in FN patients by providing a timely diagnosis and targeted antibiotic treatment.

The clinical implications of bronchoscopy for critically ill patients have been challenging to determine, with high mortality recorded in previous studies. Mortality in ICU and mechanically ventilated patients has been reported to be as high as 71% and 93% respectively by Gruson et al. [20] and 77% in mechanically ventilated patients by Rano et al. [21]. However, in these studies any association of mortality with bronchoscopy or lack thereof was not clearly elucidated. In our study, we clearly noted that the higher mortality in ICU patients was multifactorial, and fatal decline in post-bronchoscopy ventilation parameters occurred in just 14.8% mechanically ventilated patients. Interestingly, up to 50% ICU patients underwent change of treatment subsequent to BAL culture results, thus the findings of our study support timely intervention in suitable ICU patients with FN.

The predominant etiology of pneumonias was determined to be bacterial in our setting (fungal in ICU). Precedent studies largely report fungal etiologies [6], while studies reporting bacterial predominance are sparse [22]. This finding seems to suggest that institutional and local studies on etiology of pneumonia, both in ICU and non-ICU settings, are essential to improve antibiotic stewardship, and establish the relevance of our study. Cases of tuberculosis (high local endemicity of TB) in addition to hospital acquired organisms such as Stenotrophomonas and Enterococci due to prolonged hospitalization may explain this difference in our case. There was a significantly higher mortality with fungal pneumonias in our setting, in contrast to the findings of Rano et al. [21]. Interestingly, therapeutic course of antifungal therapy was given to 12 patients in our population with negative BAL cultures, mainly on physicians' discretion and supported by elevated serum (1, 3)-β-D Glucan and Aspergillus galactomannan levels. Previous studies in non-neutropenic patients also suggest a rather low sensitivity of bronchial washing culture for fungal pneumonias [18,23]. However, within the scope of our retrospective study, we are unable to comment further.

The limitations of this study remain its retrospective nature, due to which findings may have been affected by biases of treating physicians. For the same reason, the observation of increased risk of mortality in certain subsets of patients can be reported as prevalence data more than true association. None of our patients underwent transbronchial lung biopsy, which in certain precedent studies improved the diagnostic yield [12,24]. We assume that the treating pulmonologists had not opted for biopsy in order to minimize the complication risk associated with it in previous studies [12,24]. Similarly, protected specimen brush technique was not performed, however in earlier study by Boersma et al. [9], this technique did not add to the overall diagnostic yield. Despite that, the study offers an interesting and valuable insight to the role of BAL in diagnosis and management of respiratory infections in FN patients in our population setting. Several findings of this study reinforce our confidence in advocating timely intervention for both critically and non-critically ill FN patients.

## Conclusions

Flexible bronchoscopy has an appreciable diagnostic yield with BAL in FN patients with lung infiltrates. This study concludes that BAL also has an acceptable safety profile and poses minimal complication risk to patients in both ICU and non-ICU setting, and is largely beneficial in terms of facilitating judicious use of antibiotics, especially de-escalation of unwarranted empirical antibiotic therapy. This study suggests that while there may be no impact of the duration of empirical antibiotics on culture yield of BAL, longer duration of neutropenic fever as well as hospitalization and empirical treatment prior to bronchoscopy may result in poorer outcomes in such patients. This reiterates the approach for early diagnostic imaging and intervention for FN patients. The predominant etiology of infection was determined to be bacterial in our population setting, whereas fungal pneumonias were determined to be associated with significantly higher mortality. Of note, among critically ill patients, especially those maintained on mechanical ventilation, the risk of bronchoscopy-related complications is much lower than the actual mortality.
